# A Case of “Late” Postsurgical Hypoparathyroidism

**DOI:** 10.1155/2017/3962951

**Published:** 2017-05-31

**Authors:** Cesar Augusto Simões, M. K. Costa, L. B. Comerlato, A. A. Ogusco, V. Araújo Filho, R. A. Dedivitis, C. R. Cernea

**Affiliations:** ^1^Department of Head and Neck Surgery, University of Santo Amaro School of Medicine, São Paulo, SP, Brazil; ^2^University of Santo Amaro School of Medicine, São Paulo, SP, Brazil; ^3^Department of Head and Neck Surgery, Hospital das Clínicas, University of São Paulo School of Medicine, São Paulo, SP, Brazil

## Abstract

**Introduction:**

Postsurgical hypoparathyroidism normally occurs a short time after thyroid surgery in form of two clinical syndromes of different etiology and prognosis. The first is transitory and might spontaneously recover within a few weeks or months. The second is permanent and needs a definitive treatment. Only few cases of hypoparathyroidism clinically evident after many years from surgery have been reported.

**Case Report:**

A case of hypoparathyroidism clinically evident only three and a half years after surgery is reported. Our findings and review of a few cases reported by medical literature suggest the existence of a third form of postsurgical hypoparathyroidism, characterized by a late beginning.

## 1. Introduction

Hypocalcemia is one of the most frequent complications after thyroidectomy and is related to the accidental removal of the parathyroid glands, vascular pedicle damage, extensive surgical procedure, and hyperthyroidism [1, 2]. It is traditionally classified into permanent (up to 33% of the cases) and transient hypoparathyroidism (up to 87% of the cases) [1]. At first, the symptoms appear within 48 hours and persist for a period of less than 6 months. In the second case, the symptoms remain for up to six months [1].

A rare clinical presentation has been reported in the literature, in which hypoparathyroidism manifests some months or years after thyroid surgery, being referred to as “late hypoparathyroidism” [3]. This form can be associated with other causes of nonsurgical hypoparathyroidism, with etiology being divided into poor development of parathyroids, destruction of parathyroids, the control of peripheral resistance to PTH secretion, vitamin D deficiency, medication, hyperphosphatemia, pancreatitis, accelerated bone mineralization, and septicemia [2].

The purpose of this article is to report a late case of hypocalcemia after 42 months of total thyroidectomy in patients with serious disease which showed hypocalcemia.

## 2. Case Report

**Figure 1:**
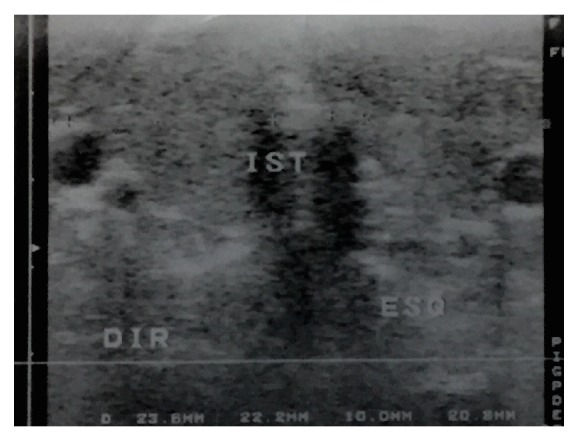
Ultrasound before thyroidectomy shows heterogeneous parenchyma with an estimated size of 45 cm^3^.

A single 24-year-old woman, with diagnosis of Grave's disease diagnosed 15 years before, was in continuous use of methimazole 80 mg a day. At the physical examination, the thyroid gland was increased by 3 times the normal size and with nodules on both sides without any sign or symptom of hypocalcemia. Thyroid hormones, serum calcium, vitamin D phosphorus, and PTH were normal and ultrasound showed heterogeneous parenchyma with an estimated size of 45 cm^3^(Figure 1). The patient underwent total thyroidectomy in May, 2012, with a surgical pathology report diagnosing a multinodular goitre, without the evidence of parathyroid glands.

In the postoperative period, besides the levothyroxine replacement, calcium carbonate 1 g a day was prescribed, due to the signs and symptoms confirmed in laboratory tests of hypoparathyroidism. After 2 months, with tests showing insufficient 25-hydroxyvitamin-D 25 ng/mL with total serum PTH and normal calcium, we prescribed vitamin D 1.000 IU a day and for 5 months after the operation in order that the patient can develop total clinical and laboratorial improvement, and we discontinued calcium intake; however, prescription of vitamin D was kept for over 4 months.

By the end of 2015, 42 months after the thyroidectomy, she presented daily crises of cramps in the upper limbs with duration of up to 3 hours each episode having the clinical and laboratory diagnosis of hypocalcemia. Total calcium level was 3 mg/dL (normal values, 8.6–10.2 mg/dL) and PTH was 3.1 pg/mL (normal values, 15–65 pg/mL), with 25-vitamin D-OH 32 ng/mL (normal values up to 30 ng/mL). Calciferol 0.5 mg a day and calcium carbonate 2 g a day were immediately prescribed.

After 2 months, the symptoms were successfully treated and normal tests were achieved, keeping use of calciferol and calcium in the same dosages and quarterly control assessments until the present time.

## 3. Discussion

Unlike the cases already reported in the literature [3], the patient presented hypocalcemia applicant after 42 months of the total thyroidectomy. There were no other associated diseases that could justify the hypoparathyroidism [2, 4].

In surgical manipulation during thyroidectomy, inadvertent resection or injury of vascular pedicle of the parathyroid glands can occur, with sudden and significant reduction in the levels of parathyroid hormone, which would lead to parathormone-dependent hypocalcemia [1], which probably causes the transitory hypoparathyroidism. One possible explanation for such a late event can be the scar retraction of parathyroids already handled and hypovascularized in the surgical bed with consequent worsening of hypovascularization.

Another predictor factor closely related to hypoparathyroidism is the decreasing levels of vitamin D [5]; however, in the most recent monitoring, the patient always presented normal serum levels without using exogenous vitamin. Thus, vitamin D insufficiency and Graves' disease could be considered predictive factors for the late hypoparathyroidism. However, further studies should be conducted to confirm this hypothesis.

We report the case of hypocalcemia belated only after three and a half years of thyroidectomy in patients with Graves' disease and transitory hypoparathyroidism, suggesting the existence of another mode of hypoparathyroidism after thyroidectomy.
